# Navigating the Complexity of Thalamic Abscess: A Systematic Review of Case Studies and Evidence-Based Management

**DOI:** 10.7759/cureus.71468

**Published:** 2024-10-14

**Authors:** Injam Ibrahim Sulaiman, Younus M Al-Khazaali, Rania H Al-Taie, Sajjad G Al-Badri, Mustafa Ismail

**Affiliations:** 1 Department of Surgery, College of Medicine, Hawler Medical University, Erbil, IRQ; 2 Department of Surgery, College of Medicine, Al-Nahrain University, Baghdad, IRQ; 3 Department of Surgery, College of Medicine, University of Mustansiriyah, Baghdad, IRQ; 4 Department of Surgery, College of Medicine, University of Baghdad, Baghdad, IRQ; 5 Department of Surgery, Baghdad Teaching Hospital, Medical City Complex, Baghdad, IRQ

**Keywords:** abscess drainage, brain abscess, neurosurgery, stereotactic aspiration, thalamus abscess.

## Abstract

Thalamic abscesses are rare, life-threatening conditions and represent only a few percent of the total cases of brain abscesses. Due to their deep location and critical involvement of sensory and motor pathways, they remain one of the most challenging entities to diagnose and manage. Despite advances in neuro-imaging and neurosurgical techniques, thalamic abscess continues to be a challenge or question in clinical practice regarding optimum treatment modality. Given the nature of the description, a systematic review was performed according to the Preferred Reporting Items for Systematic Reviews and Meta-Analyses (PRISMA) guidelines. Case reports were retrieved after extensive searches in PubMed and Scopus with specified inclusion criteria: isolated thalamic abscesses that needed surgical interventions. The reporting quality was assessed according to CARE (CAse REports) guidelines, while data on clinical presentation and diagnostic approach were extracted.

Thirty-three cases with the diagnosis of thalamic abscess were reviewed. The most common presentations included headache, hemiparesis, and altered sensorium. CT and MRI were the common diagnostic tools for these patients; stereotactic aspiration was the most common surgical intervention performed. *Streptococcus* species were the most common causative organisms. At follow-up, the majority of patients had a good outcome with complete or near-complete recovery. There were rare complications, such as hydrocephalus and recurrence, and mortality was low. Thalamic abscesses are infrequent but have a good prognosis in case of appropriate diagnosis and treatment with stereotactic aspiration and appropriate antibiotic therapy. The present systematic review points out the need for adopting an individual approach to treatment and for further studies that could provide better information on diagnostic and therapeutic strategies regarding this severe disease.

## Introduction and background

Thalamic abscesses, although relatively rare, constitute approximately 1.3-6% of all brain abscesses. Their central location within the brain parenchyma poses a significant challenge, often leading to delayed diagnosis or complications such as intraventricular rupture, which result in high morbidity and mortality [1]. They occur from direct extension sites like sinusitis and hematogenous spread from other infected foci, which are often far away and involve organisms such as *Streptococcus* species [2].

The predominant pathogens implicated in the pathogenesis of thalamic abscesses are the *Streptococcus* species and, more importantly, the *Streptococcus anginosus *group due to their strong tendency to induce abscess formation by the production of toxins and through deploying immune evasion strategies. Secondly, conditions may generally predispose them to such infections, which include but are not limited to diabetes mellitus, malignancy, and hereditary disorders such as hereditary hemorrhagic telangiectasia (HHT) [1,2].

Thus, the diagnosis of thalamic abscesses has been pretty difficult due to their deep intracranial location and nonspecific clinical presentations ranging from altered consciousness to focal neurological deficits, including hemiparesis or aphasia. Neuroimaging provides important support for their diagnosis and their differential diagnosis from other pathologies within the cranial vault, such as tumors or even infarctions, by means of neurological intervention with enhancement through computed tomography (CT) and magnetic resonance imaging (MRI). Treatment most frequently involves neurosurgical involvement in the form of stereotactic aspiration, usually with prolonged antibiotic therapy.

Although there is also a more conservative way of using only antibiotics that have been moderately successful, as seen in selected cases where surgery is dangerous [1], thalamic abscesses are now being better managed with advances in neuroimaging/neurosurgical techniques. The introduction of stereotactic surgery was, therefore, a breakthrough in the history of accessing difficult areas of the brain and has remained, up to now, a preferred method in the treatment of thalamic abscesses. Recently, stereotactic techniques such as CT/MRI-guided aspiration have been developed to include being minimally invasive with minimal complication rates. These procedures allow for the drainage of purulent material with minimum risk of damaging critical structures of the brain, thereby reducing mass effect [3].

Stereotactic surgery thus allows for precision in the least invasive manner, which improves the outcome in patients with deep-seated brain lesions; hence, treatment modalities for thalamic abscess, with newer advances in imaging and robotic-assisted techniques, are evolving to provide hope for improved morbidity and mortality rates. The present systematic review critically evaluates the available literature regarding the pathophysiology and clinical presentation of thalamic abscesses, the challenges in diagnosis, and the therapeutic approaches that have been encountered. In this respect, the current review will try to bring forward new knowledge about the etiology and outcomes of thalamic abscesses, the role of modern neuroimaging and stereotactic surgical techniques, as well as identify the best optimal treatment strategy that would improve patient outcomes.

## Review

Methods

**Figure 1:**
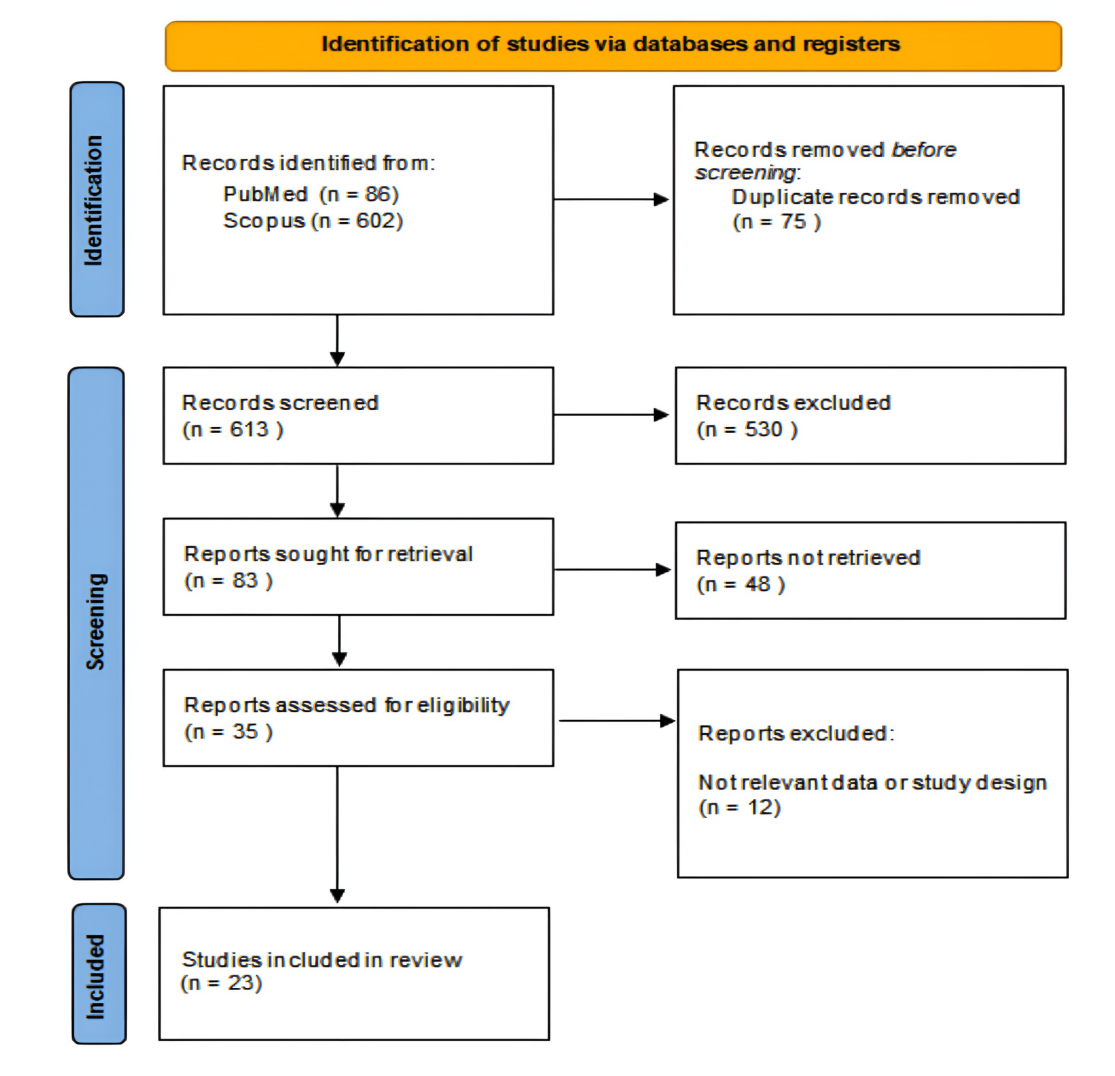
PRISMA flowchart of the included studies. PRISMA: Preferred Reporting Items for Systematic Reviews and Meta-Analyses

This systematic review was conducted following the Preferred Reporting Items for Systematic Reviews and Meta-Analyses (PRISMA) guidelines (Figure 1) [4], ensuring a transparent and replicable approach.

Search Strategy

A literature search was carried out using the Scopus and PubMed databases. The search terms included "thalamic" AND "abscess," There was no restriction on the date of publication, but articles were limited to those published in the English language. This would ensure that the search was as inclusive as possible and focused on studies relevant to the research question.

Inclusion and Exclusion Criteria

To preserve relevance and quality in the studies that would be included, specific inclusion and exclusion criteria were considered. Every case report included involved an isolated thalamic abscess, meaning a thalamic abscess unassociated with other brain lesions. Likewise, case reports were obtained from papers that reported surgical intervention targeting the thalamic abscess alone. Another inclusion criterion was the availability of follow-up data following such procedures to ascertain postoperative outcomes. Exclusions were multiple brain lesions, an intervention not based on surgery for the thalamic abscess, or the lack of follow-up information. Case reports also needed to have sufficient clinical detail including the data about the presentation, diagnosis, management, causing further exclusions, while studies involving non-human subjects were likewise excluded.

Study Selection

After the search, the 688 retrieved articles were imported from the databases to Rayyan, a freely available web application designed for systemic reviews (Rayyan, Cambridge, Massachusetts, United States). This software allowed blinded, independent title and abstract screening against the research question by two reviewers. Thereafter, disagreement between the reviewers was resolved through discussion, while cases of disagreement that could not be resolved by discussion were put forward to a third reviewer. Articles making it to this screening level were then progressed to full-text analysis for inclusion.

Quality Assessment

Given that all included studies were case reports, the CARE (CAse REport) guidelines were used to assess the quality of each report [5]. The CARE guidelines provided a framework for evaluating the case reports' clarity, completeness, and transparency (Table 1) [6-28]. Only those case reports that adhered to most of the CARE components were included in the final review.

**Table 1:** Quality assessment of case reports on thalamic abscess using CARE guidelines CARE: CAse REports

Study ID	Author, Year	Patient Information	Clinical Findings	Diagnostic Assessment	Therapeutic Interventions	Follow-up/Outcomes	Discussion/Conclusions	Overall Quality
1	Karageorgiou et al. (2014) [6]	Comprehensive	Detailed	Thorough	Well-documented	Well-documented	Relevant	High
2	Peker et al. (2008) [7]	Comprehensive	Detailed	Thorough	Well-documented	Well-documented	Relevant	High
3	Venger et al. (1987) [8]	Comprehensive	Detailed	Thorough	Well-documented	Well-documented	Relevant	High
4	Gupta et al. (2024) [9]	Comprehensive	Detailed	Thorough	Well-documented	Well-documented	Relevant	High
5	Mantova and Moya (2023) [10]	Comprehensive	Detailed	Thorough	Well-documented	Well-documented	Relevant	High
6	Dillen et al. (2018) [11]	Comprehensive	Detailed	Thorough	Well-documented	Well-documented	Relevant	High
7	Şenol et al. (2016) [12]	Comprehensive	Detailed	Thorough	Well-documented	Well-documented	Relevant	High
8	Ganesan et al. (2019) [13]	Comprehensive	Detailed	Thorough	Well-documented	Well-documented	Relevant	High
9	Callovini et al. (2009) [14]	Comprehensive	Detailed	Thorough	Well-documented	Well-documented	Relevant	High
10	Teixeira et al. (2010) [15]	Comprehensive	Detailed	Thorough	Well-documented	Well-documented	Relevant	High
11	Chang et al. (2010) [16]	Comprehensive	Detailed	Thorough	Well-documented (Stereotactic aspiration, Antibiotics)	Variable neurological deficits	Relevant	High
12	Gajdhar and Yadav (2005) [17]	Comprehensive	Detailed	Thorough	Well-documented	Well-documented	Relevant	High
13	Shahzadi et al. (1996) [18]	Comprehensive	Detailed	Thorough	Well-documented	Well-documented	Relevant	High
14	Yamamoto et al. (1993) [19]	Comprehensive	Detailed	Thorough	Well-documented	Not well-documented	Relevant	Moderate
15	Lutz et al. (1994) [20]	Comprehensive	Detailed	Thorough	Well-documented	Well-documented	Relevant	High
16	Megens et al. (1992) [21]	Comprehensive	Detailed	Thorough	Well-documented	Well-documented	Relevant	High
17	Mohanty et al. (1999) [22]	Comprehensive	Detailed	Thorough	Well-documented	Well-documented	Relevant	High
18	Naidu (1988) [23]	Comprehensive	Detailed	Thorough	Well-documented	Well-documented	Relevant	High
19	Broggi et al. (1985) [24]	Comprehensive	Detailed	Thorough	Well-documented	Well-documented	Relevant	High
20	Bhatia et al. (1986) [25]	Comprehensive	Detailed	Thorough	Well-documented	Well-documented	Relevant	High
21	Law et al. (1976) [26]	Comprehensive	Detailed	Thorough	Well-documented	Well-documented	Relevant	High
22	Black et al. (1983) [27]	Comprehensive	Detailed	Thorough	Well-documented	Well-documented	Relevant	High
23	Ercius et al. (1982) [28]	Comprehensive	Detailed	Thorough	Well-documented	Well-documented	Relevant	High

Data Extraction

Data extraction was performed using a predefined template to ensure consistency across studies. The key information extracted included study characteristics (such as author and year), patient demographics (age, gender, and comorbidities), the etiology and clinical presentation of the thalamic abscess, and details of the surgical intervention (including the type of surgery and whether stereotactic techniques were used). Additionally, the duration of follow-up and the patient outcomes, such as neurological recovery, complications, or mortality, were also recorded.

Data Synthesis

Due to the heterogeneity of the case reports, particularly regarding differences in patient populations, clinical presentations, and treatment strategies, a qualitative synthesis was performed. Each case was carefully analyzed in terms of its clinical course, the nature of the surgical intervention, and the outcomes. Special attention was given to the impact of surgical treatment and follow-up on patient prognosis. A quantitative meta-analysis was not feasible, given the variability in study designs and reported outcomes across the included studies.

Results

Overview of the Studies and Patient Demographics

This systematic review encompassed 23 studies detailing 33 unique cases of isolated thalamic abscesses. The patient demographics had a broad range, with ages spanning from seven to 72 years. Of these cases, 65% were male, though both genders were equally represented in more recent reports. The majority of patients were immunocompetent, though certain individuals had underlying comorbidities that may have contributed to the development of the abscess, such as congenital heart disease or prior infections. Despite the range of predisposing factors, no clear demographic pattern emerged, underscoring the unpredictable nature of thalamic abscesses across age, sex, and health status (Table 2) [6-28].

**Table 2:** Summary of clinical, diagnostic, and therapeutic data from case reports on thalamic abscess CSF: cerebrospinal fluid; DBS: deep brain stimulation; DVT: deep vein thrombosis; VP: ventriculoperitoneal; SPECT: single photon emission computed tomography; GCS: Glasgow Coma Scale

ID	Author (Year)	Study Design	Number of Cases Reported	Demographic Data	Comorbidities	Symptoms and Duration of Symptoms	Neurological Examination Findings	Diagnostic Modalities and Findings	Abscess Location	Etiology of the Abscess	Surgical and Non-Surgical Treatment	Immediate Post-operative Complications and Long-term Complications	Follow-up Duration and Findings	Radiological Outcome	Clinical Outcome	Recurrence
1	Karageorgiou et al. (2014) [6]	Case report	1	56-year-old Caucasian male	No significant comorbidities reported	Five days of persistent headache, malaise, fever	Drowsy but rousable, GCS 14/15, left-sided past-pointing and intention tremor, unsteady gait	CT showed 28 mm low-density lesion in right basal ganglia, MRI showed a 30 mm ring-enhancing mass in the right thalamus	Right thalamus, 30 mm with surrounding edema and 4 mm midline shift	Streptococcus milleri	Stereotactic puncture, external drainage, Ommaya reservoir insertion, intrathecal and systemic antibiotics	Pus re-accumulation requiring re-aspiration, intraventricular rupture with spread to CSF, cerebellar infarct from septic embolus	2 months; complete radiological resolution	Complete resolution	Full recovery with no residual neurological deficit	None
2	Peker et al. (2008) [7]	Case report	1	14-year-old female	No significant comorbidities reported	One week of left hemiparesis and headache	Mild left hemiparesis, mild hemihypoesthesia, mildly hyperreflexic deep tendon reflexes	CT and MRI revealed a ring-enhancing mass in the right thalamus	Right thalamus	*Peptostreptococcus *species	Stereotactic drainage, antibiotic treatment (ceftriaxone, metronidazole), later DBS	Development of Holmes' tremor four months later requiring DBS	2.5 years; 90% reduction in tremor symptoms	Abscess drained successfully	Marked improvement, with a 90% reduction in tremor	None reported
3	Venger et al. (1987) [8]	Case report	1	66-year-old male, physician	The patient had a history of influenza prior to developing symptoms, but no chronic conditions were mentioned.	Headache, left-hand numbness and weakness	Left hemiparesis, sensory loss	CT showed a ring-enhancing mass in the right thalamus	Right thalamus	Histoplasma capsulatum	Ultrasonically guided biopsy, amphotericin treatment	Mild hydrocephalus	15 months	Complete resolution of abscess	Full recovery with minor sensory deficit	None
4	Gupta et al. (2024) [9]	Case report	1	39-year-old male, laborer	No significant comorbidities reported	Headache, vomiting, altered sensorium	GCS 10/15, right hemiparesis	CT: left thalamic abscess	Left thalamus	Rhinocladiella mackenziei	VP shunt, craniotomy, antifungal (voriconazole)	Hydrocephalus treated, hemiparesis persists	9 months	Abscess resolved after treatment	Good recovery with mild hemiparesis	None
5	Mantova and Moya (2023) [10]	Case report	1	46-year-old female, immunocompetent	No significant comorbidities reported	Six-day headache, vomiting, photophobia, unsteady gait	Ataxic gait, photophobia, normal pupils	MRI showed a thalamic abscess	Right thalamus	Streptococcus intermedius	Surgical aspiration and antibiotics	None reported	2 months	Complete resolution	Full recovery	None
6	Dillen et al. (2018) [11]	Case report	1	39-year-old female	The patient had a history of hypertension.	Fever for two weeks, unresponsive on arrival	Extensor posturing, fixed dilated pupil, left hemiplegia	MRI confirmed right thalamic abscess	Right thalamus	Streptococcus intermedius	Apollo suction device for drainage, VP shunt, antibiotics	Pulmonary embolism, DVT requiring thrombectomy, IVC filter	3 months	Abscess resolved on imaging, hydrocephalus treated with shunt	Minor residual hemiparesis, significant neurological recovery	None
7	Şenol et al. (2016) [12]	Case report	1	25-year-old male	No significant comorbidities reported	Headache, fever, right hemiparesis	Right-sided hemiparesis	MRI showed ring-enhancing left thalamic abscess	Left thalamus	Streptococcus constellatus	CT-guided stereotactic aspiration and antibiotic therapy	None	12 weeks	Complete resolution	Full recovery	None
8	Ganesan et al. (2019) [13]	Case report	1	42-year-old male	No significant comorbidities were mentioned.	Headache, vomiting, diminished sensation on right side	Sensory impairment of the right half of body including face	MRI showed left thalamic ring-enhancing lesion, CT showed ring-enhancing lesion with surrounding edema	Left thalamus	Streptococcus sanguinis	Craniotomy, transventricular excision	None reported	2 months	Abscess fully excised, resolved	Full recovery, neurologically stable	None
9	Callovini et al. (2009) [14]	Case series and literature review	3	Adults aged 32, 68, and 72 years	One patient had a history of diabetes.	Headache, cognitive impairment, hemiparesis	Altered consciousness, GCS 10-12, hemiparesis, drowsiness	CT, MRI revealed ring-enhancing lesions in the thalamus	Thalamus (various sides)	Staphylococcus aureus, Listeria monocytogenes	Stereotactic aspiration, catheter drainage, intrathecal and systemic antibiotics	No significant complications, one case of mild motor deficits	4-5 years	Complete resolution in two, encapsulated area in one	Two full recoveries, one mild disability	None
10	Teixeira et al. (2010) [15]	Case report	1	32-year-old male	The patient had congenital heart disease.	Headache, visual impairment, hemiparesis, somnolence	Right hemiparesis, disoriented, papilledema	CT scan revealed left thalamic cystic mass	Left thalamus	Paracoccidioides brasiliensis	Stereotactic drainage, intralesional amphotericin-B, systemic antibiotics	Initial deterioration, then improved with amphotericin-B	10 years	Complete recovery, no recurrence	Full neurological recovery	None
11	Chang et al. (2010) [16]	Retrospective review (8 cases)	8 (two of them in the left thalamus)	Age range 41–80, mean age 61, 75% males	Case 1: This patient had a history of previous neurosurgery (craniectomy for intracerebral hemorrhage). Case 2: No comorbidities were mentioned for this patient.	Case 1: Symptoms: Right hemiparesis, dysarthria, and altered mental status. Case 2: Symptoms: Left hemiparesis, headache, and altered sensorium.	Hemiparesis common, sensory impairment	MRI, CT of deep-seated abscesses	Left Thalamus	Case 1: *Klebsiella pneumoniae*; Case 2: *Streptococcus viridans*	Stereotactic aspiration, ventriculoperitoneal shunt, antibiotics	Case 1 : The patient died 10 days after surgery due to complications from a pulmonary embolism. Case 2:The patient survived but was left with persistent right hemiparesis after treatment.	Case 1 : The patient died 10 days after surgery due to complications from a pulmonary embolism. Case 2:The patient survived but was left with persistent right hemiparesis after treatment.	Complete resolution in surviving case	Complete resolution in surviving case	None noted
12	Gajdhar and Yadav (2005) [17]	Case report	1	7-year-old girl, cyanotic heart disease	Cyanotic heart disease	Fever, headache, vomiting, altered sensorium, left hemiparesis	GCS 13/15, worsening to GCS 11/15	CT showed a right thalamic abscess	Right thalamus	Streptococcus viridans	Endoscopic aspiration, antibiotics (gentamycin), intraventricular drain	No significant complications	1 year	Complete disappearance of abscess	Full recovery	None
13	Shahzadi et al. (1996) [18]	Case series (20 cases)	1	21-year-old male	Hemiparesis, fever, vomiting	He had a postoperative infection as the source of the abscess	GCS < 8, right hemiparesis	CT/MRI showed right thalamic abscess	Right thalamus	Haemophilus influenzae	Stereotactic aspiration, antibiotics	None reported	Multiple months	Complete resolution	Full recovery	None
14	Yamamoto et al. (1993) [19]	Case reports (2 cases)	2	Case 1: 9-year-old girl, congenital heart disease; Case 2: 30-year-old female	Case 1 (9-year-old girl): The patient had congenital heart disease (single ventricle with pulmonary stenosis), which predisposed her to the development of a brain abscess. Case 2 (30-year-old female): No significant comorbidities were mentioned for this patient.	Case 1: Headache, vomiting, hemiparesis; Case 2: Headache, vomiting, hemiparesis	Case 1: Hemiparesis, coma; Case 2: Hemiparesis, neglect	CT: ring-enhanced lesions	Case 1: Right thalamus; Case 2: Left thalamus	Case 1: Culture negative; Case 2: *Peptococcus*	Case 1: Burr hole aspiration; Case 2: Stereotactic aspiration, antibiotics	Case 1: Died post-op due to heart failure; Case 2: None	Case 1: None; Case 2: 3 years	Case 1: None; Case 2: Abscess fully resolved	Case 1: Death; Case 2: Full recovery	None
15	Lutz et al. (1994) [20]	Case series (3 cases)	3 (2 of them had Thalamic abscess)	Case 1: A right-handed female born in 1953 (41 years old). Case 2: A 58-year-old right-handed female, a retired pediatric nurse.	Case 1: The patient had Tetralogy of Fallot, with pulmonary hypoplasia and collateral bronchial circulation since birth. Case 2:This patient had recently undergone dental extraction (molar tooth), which likely contributed to the abscess formation.	Headache, vomiting, altered consciousness	Hemiparesis, GCS 13-14, altered consciousness	CT-guided stereotactic puncture	Case 1: Between the left thalamus and capsula interna, compressing the 3rd ventricle and producing a left Monro blockade Case 2: Left thalamus with perifocal low density and a Monro blockade	Case 1: *Peptostreptococcus micros; *Case 2: *Streptococci Milleri*	Stereotactic puncture, drainage, systemic antibiotics	Ventricular rupture in 2 cases, treated with VP shunt	5 years	Full resolution in 2 cases,	Full recovery in 2	None
16	Megens et al. (1992) [21]	Case report	1	33-year-old male	History of infected dental caries	Headache, vomiting, speech difficulty, right-hand clumsiness	Subcortical aphasia, right hemiparesis	CT: left thalamic abscess, SPECT showed hypoperfusion in cortex	Left thalamus	Streptococcus milleri	Stereotactic aspiration, antibiotics (penicillin, metronidazole)	None reported	7 months	Abscess resolved, SPECT showed improved perfusion	Full recovery	None
17	Mohanty et al. (1999) [22]	Case series (3 cases)	3	Males aged 19-25	Case 1 (25-year-old male): The patient had pulmonary tuberculosis and was on antituberculous treatment (ATT) for seven months before presenting with the thalamic abscess. Case 2 (20-year-old male): The patient had a history of pulmonary tuberculosis, treated 10 years prior with a full course of isoniazid, rifampicin, pyrazinamide, and streptomycin.	Progressive hemiparesis, headache, vomiting	Papilledema, hemiparesis	CT: multiloculated lesions with central hypodensity, peripheral rim enhancement	Thalamus (2 cases)	Mycobacterium tuberculosis	Stereotactic aspiration, antituberculous therapy	Hydrocephalus in 1 case, treated with VP shunt	1 year	Complete resolution in 2 cases, small residual lesion in 1	Neurological improvement, no major deficits	None
18	Naidu (1988) [23]	Case report	1	32-year-old male with cyanotic heart disease	Cyanotic heart disease	Fever, headache, vomiting, convulsions	Drowsiness, right hemiparesis, facial nerve paresis	CT: hypodense lesion in left thalamus	Left thalamus	Culture negative	Burr hole aspiration, antibiotics	Hydrocephalus requiring VP shunt	1 year	Complete resolution of abscess, ventricles reduced	Full recovery with no major deficits	None
19	Broggi et al. (1985) [24]	Case series (4 cases)	4 (one patient with a thalamic abscess)	45-year-old male	Non-Reported.	Rapidly increasing left hemiparesis and headache, while mild fever was present without haematochemical signs of infection	Consciousness disturbances, hemiparesis	CT scan showed ring-enhancing lesions	Right Thalamus	Immobile cocchi chains	Stereotactic implantation of catheter with antibiotic therapy	None reported	45 months	Complete resolution	Full recovery in all cases	None
20	Bhatia et al. (1986) [25]	Case series	4	Case 1: 30-year-old woman. Case 2: 18-year-old male. Case 3: 28-year-old male (renal transplant). Case 4: 30-year-old male	Case 3: Renal transplant, immunosuppressants Case 4: Untreated cervical lymphadenopathy	Case 1: Fever, headaches for 10 days. Case 2: Severe headache, loss of consciousness, right hemiparesis. Case 3: Headache, fever, progressive left hemiparesis. Case 4: Headache, vomiting, drowsiness for 10 days	Case 1: Papilledema, neck stiffness. Case 2: Right hemiparesis, cerebellar signs. Case 3: Left hemiparesis, hemianopsia, cerebellar signs. Case 4: Right third nerve paresis, left spastic hemiparesis	Case 1: CT: lesions in left thalamus and frontal cortex. Case 2: CT: left thalamus, ring enhancement. Case 3: CT: right thalamus, displacement of ventricle, hydrocephalus. Case 4: CT: right thalamus, surrounding edema	Case 1: Left thalamus and frontal cortex. Case 2: Left thalamus. Case 3: Right thalamus. Case 4: Right thalamus	Case 1: Tuberculoma Case; 2: Tuberculoma; Case 3: *Mycobacterium tuberculosis*; Case 4: Tuberculoma	Case 1: Streptomycin, INH, rifampin Case 2: INH, streptomycin, thiacetazone Case 3: Stereotactic aspiration, antituberculous therapy Case 4: Streptomycin, INH, rifampin, thiacetazone	Case 4: Status epilepticus, phenytoin toxicity	Case 1: 14 months, asymptomatic Case 2: 13 months, residual calcification Case 3: Small residual lesion Case 4: Residual mass	Case 1: Resolution of thalamic tuberculoma Case 2: Smaller ring lesion Case 3: Residual lesion Case 4: Unresolved mass	Case 1: Asymptomatic Case 2: Residual calcification Case 3: Improvement Case 4: Residual hemiparesis	None
21	Law et al. (1976) [26]	Case series	1	55-year-old male	None	Cough, fever, chills, anorexia for 6 weeks	Right lower facial weakness, right plantar response	Angiography showed avascular mass in the left thalamus	Left thalamus	Streptococcus	Subtotal excision	None reported	5 years, asymptomatic	Normal post-op	Asymptomatic	None
22	Black et al. (1983) [27]	Case report	1	63-year-old female	Chronic bronchitis, treated tuberculosis	Hemoptysis, chronic cough, bifrontal headache, fever, meningismus, hemiplegia	Right facial weakness, right hemiplegia, hyperreflexia, right Babinski sign, dysphasia	CT scan showed a left thalamic enhancing mass, followed by ventriculitis and hydrocephalus	Left thalamus	Streptococcus	Aspiration of 15 mL of pus, followed by intravenous penicillin, ventriculoperitoneal shunt	Shunt malfunction, dysphasia recurrence	9 months, neurologically normal after shunt revision	Normal ventricular size on follow-up CT	Full recovery after shunt revision	Dysphasia recurrence after shunt malfunction
23	Ercius et al. (1982) [28]	Case report	1	42-year-old male	None reported	Left hemiparesis, headache	Dense left hemiparesis	CT: ring-enhancing lesion in right thalamus	Right thalamus	Alpha hemolytic streptococci, *Eubacterium lentum*	Transcallosal drainage, antibiotics	None reported	1 year	Complete resolution	Full recovery with mild left-sided paresis	None

Clinical Presentation and Neurological Findings

The clinical presentation of thalamic abscesses varied but shared some key characteristics across cases. The most common symptoms included headache, hemiparesis, fever, and altered consciousness. For example, Karageorgiou et al. documented a 56-year-old man with a five-day history of headache and malaise, accompanied by neurological deficits such as left-sided intention tremor and unsteady gait [6]. Like many other cases, this patient presented with focal neurological symptoms reflective of the involvement of the thalami in both motoric and sensory pathways. In the case reported by Peker et al., it was a 14-year-old girl who had been presenting for one week with left hemiparesis and headache, an indication of early thalamic abscess development [7]. In a more critical case, Gupta et al. referred to a patient who already showed a GCS of 10/15 with right hemiparesis, a sign that such abscesses can also present with severe cognitive and motor impairments [9]. There were unique neurological presentations. The patient from Peker et al. developed a Holmes's tremor due to stereotactic drainage, which is a rare neurological disorder characterized by slow, coarse tremors [7]. This illustrates the variety of sequelae, sometimes complex, attendant to thalamic infections. Neurological impairments across cases ranged from mild sensory loss to profound motor deficits and altered mental states, as seen in a case reported by Dillen et al., in which a 39-year-old female patient had fixed dilated pupils and extensor posturing, indicative of severe intracranial pathology [11].

Diagnostic Modalities and Abscess Localization

Neuroimaging thus formed the cornerstone for the diagnosis of thalamic abscesses in all cases. CT and MRI consistently disclosed a ring-enhancing lesion within the thalamus. Karageorgiou et al. reported a case of a right thalamic ring-enhancing mass measuring 30 mm, confirmed by MRI [6]. Likewise, Venger et al. made the same diagnosis in their case of a 66-year-old male physician [8]. As seen in the case presented by Gupta et al., there was a left-sided thalamic abscess evident on axial CT scans and further confirmed by MRI studies [9]. The latter explained how imaging provides insight into the exact site and size of the abscess. Indeed, the overall imaging studies usually demonstrated secondary findings, such as surrounding edema and midline shifts. These are signs suggestive of an increase in intracranial pressure and poor clinical presentation.

Whereas most of the abscesses were located in the right thalamus, several instances were found to have a left thalamic abscess, as described by Şenol et al. [12] and Ganesan et al. [13]. This apparently did not alter whether the clinical course or outcome would be severely affected since there has been consistent anatomical involvement of the thalamus with remarkable motor and sensory deficits.

Etiological Agents

Nowadays, several pathogens have been implicated in the development of thalamic abscesses, most of them being *Streptococcus* species. For example, in the paper by Karageorgiou et al., it was *Streptococcus milleri *that was isolated [6], while Mantova and Moya reported *Streptococcus intermedius* in their case [10]. In fact, this is due to the ability of organisms to thrive in environments with low oxygen levels and their capacity to evade immune detection. Other rare bacteria, such as *Peptostreptococcus*, and fungal organisms, like *Rhinocladiella mackenziei* in Gupta et al. [9], were also detected. Some of these reports implicate fungi, such as those by Venger et al. [8] and Teixeira et al. [15], which describe *Histoplasma capsulatum* and *Paracoccidioides brasiliensis*, respectively. These infections bring into consideration both bacterial and fungal etiologies, especially in patients from endemic regions or with underlying immunosuppression.

Until recently, some cultures remained negative despite extensive work, which again underlined the difficulties in extracting the pathogens from the abscesses located deeply in the brain. That was the situation in cases such as that of Naidu [23], where even with sophisticated diagnosis, no pathogens were isolated.

Surgical and Medical Interventions

Generally, the thalamic abscesses were usually treated surgically; hence, the most common approach was stereotactic aspiration. In Karageorgiou et al., stereotactic puncture and external drainage were initially performed, followed by the insertion of an Ommaya reservoir for the instillation of antibiotics [6]. Stereotactic techniques allowed precise drainage of the abscess with minimal destruction of the surrounding brain tissue. Any major lesions of the thalamus are not tolerated because this organ is very important in the mechanisms of senses and motion. Peker et al. [7] and Mantova and Moya [10] used stereotactic drainage; in both cases, the abscess was resolved.

Sometimes, the surgical approaches need to be more aggressive. Thus, in Gupta et al., surgery with craniotomy was needed due to the abscess complexity with associated hydrocephalus [9], whereas Venger et al. treated a fungal abscess by *H. capsulatum* with ultrasonically guided biopsy and with amphotericin B therapy [8]. Similarly, Teixeira et al. also performed stereotactic drainage followed by intralesional amphotericin-B for the *Paracoccidioides brasiliensis* infection in their patient [15]. These cases highlight the importance of specific surgical and medical treatments depending on both the pathogen causing the infection and the complexity of the abscess.

In all instances, there was a need for postoperative antibiotic therapy. This usually consisted of broad-spectrum antibiotics such as ceftriaxone and metronidazole. Antifungal therapy included voriconazole and amphotericin-B, which was started post-drainage when the causative organisms were fungal. Antituberculous therapy was also used in the case of tuberculous abscess. For instance, Mohanty et al. reported a stereotactic aspiration followed by a full course of antituberculous drugs [22].

Complications and Outcomes in the Postoperative Period

The most significant post-surgical complication that arose in most of the cases was hydrocephalus. In a report by Karageorgiou et al., the most significant complication developed due to intraventricular rupture, leading to cerebellar infarction because of septic emboli that required further interventions [6]. Gupta et al. managed postoperative hydrocephalus with a ventriculoperitoneal shunt; however, the patient was left with residual hemiparesis [9]. More importantly, the patient of Peker et al. presented the rare and debilitating condition of Holmes' tremor four months postoperatively that required deep brain stimulation in its management [7].

Despite such complications, the overall course of the long-term prognosis remained relatively favorable. Most patients, such as Mantova and Moya, showed complete radiological and clinical recovery [10]. Even when complications arose, as demonstrated by Venger et al. [8] and Dillen et al. [11], patients eventually received significant neurological recoveries with some residual deficits.

Radiological and Clinical Outcomes

Most of them were followed up radiologically for complete resolution of the abscess, with follow-up starting from two months to 10 years. Karageorgiou et al. followed up on the complete resolution of imaging within two months [6]. Similarly, Peker et al. reported a 90% reduction in the symptoms of the tremor in a follow-up period of 2.5 years [7]. Gupta et al. also reported complete radiological resolution after nine months [9]. The patient had some residual motor deficits postoperatively.

Clinically, most of the patients had full recovery. In the cases reported by Mantova and Moya [10] and Ganesan et al. [13], patients recovered completely with no neurological deficit, while the patient in the case reported by Gupta et al. had a good recovery, notwithstanding mild hemiparesis [9]. The patient in Peker et al.'s case, who developed Holmes' tremor, showed marked improvement after DBS, with a 90% reduction in the severity of the tremor [7].

Mortality and Recurrence

Mortality was minimal, with two deaths registered. In the case reported by Teixeira et al., the patient died due to a pulmonary embolism on the 10th postoperative day [15], while in Yamamoto et al.'s case, the patient died due to heart failure not related to the abscess itself [19]. No recurrence was documented during follow-up, implying that with proper surgical and medical intervention, the long-term prognosis is good.

Discussion

**Figure 2:**
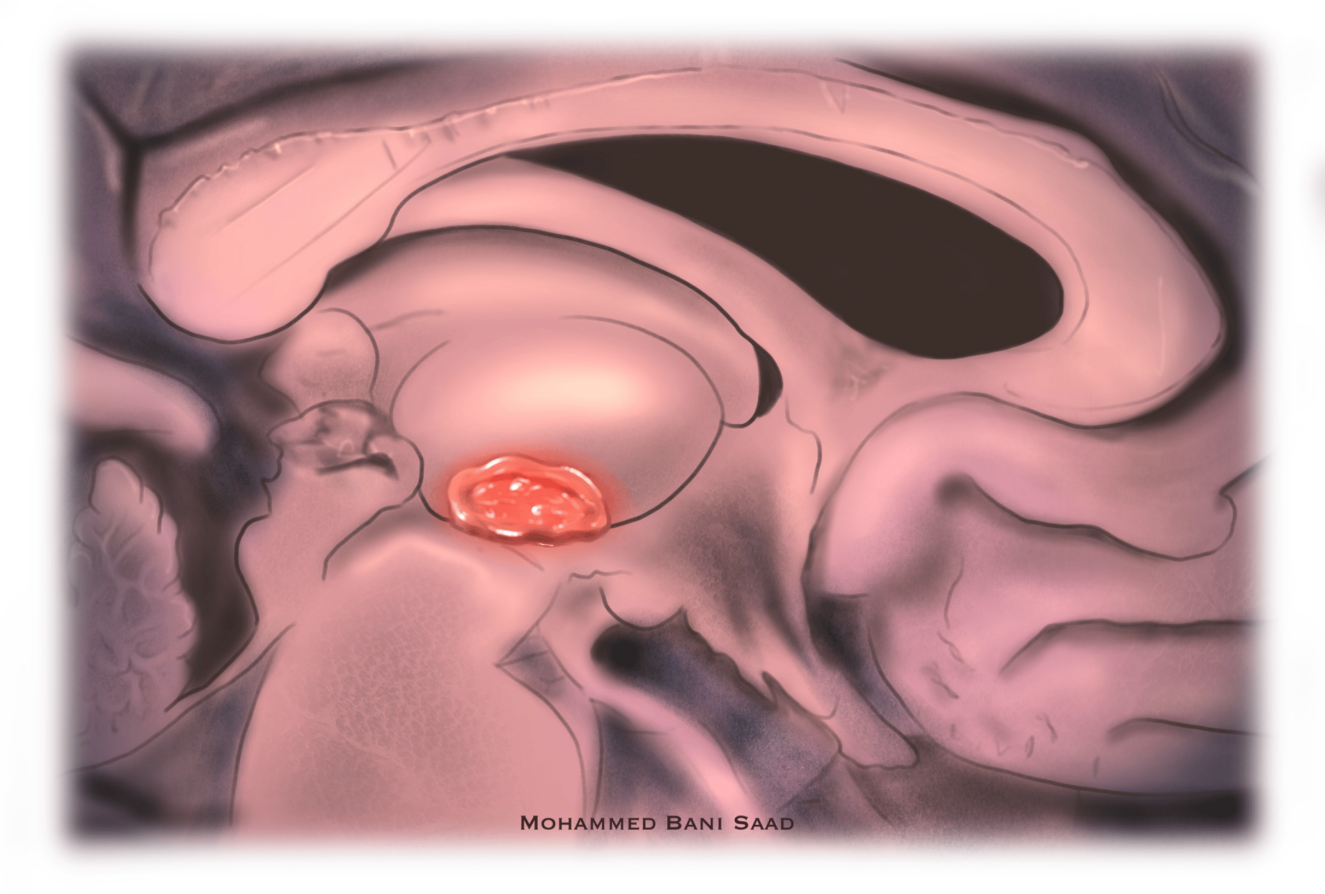
Artistic presentation of thalamic abscess Image Credit: Dr. Mohammed A. Bani Saad; specifically created for this review article

The thalamus, a vital mass of subcortical gray matter situated within the lateral walls of the third ventricle, serves as a fundamental relay station in the brain (Figure 2).

Its role extends far beyond simple sensory transmission; the thalamus integrates sensory, motor, cognitive, and emotional processes through intricate reciprocal connections with the cerebral cortex, the amygdala, and the basal ganglia. Over the last two centuries, advances in neuroanatomical techniques have enabled a finer understanding of the thalamus. Initially, early parcellation schemes based on basic histological staining revealed only a handful of nuclei. However, modern concepts that include cytoarchitecture, myeloarchitecture, and genoarchitecture have emphasized the complexity of the thalamus beyond earlier thought [29].

The problem with intracranial abscesses is that delayed diagnosis and limited access to advanced imaging significantly hinder appropriate medical intervention in most developing countries. These are usually due to bacterial or fungal infections; thus, if left unmanaged, the abscess may rapidly increase in size and intracranial pressure, leading to severe neurological deficits. These include non-specific early symptoms, such as headache and fever, making early diagnosis of utmost importance. Despite improvements in surgical and antibiotic treatments, morbidity and mortality remain high due to drug resistance and other complications such as hydrocephalus. In resource-poor settings, rapid diagnosis, timely surgical intervention, and appropriate antibiotic regimens play a complementary role in an effort toward better outcomes and reduced risk of fatality. Addressing diagnostic challenges and antibiotic resistance is bound to be important for better management of this life-threatening condition [30].

Thalamic abscesses, though rare, are a big challenge both from the point of view of diagnosis and treatment due to their deep-seated location and the great importance of the thalamus regarding motor and sensory function. The mainstay of this systematic review of 36 cases will be to impress a few important aspects related to thalamic abscesses, such as varied clinical presentation, diagnostic difficulties, diversity in etiological agents, and efficacy of modern surgical techniques that improve the outcomes of patients. The clinical presentation of thalamic abscesses varies from mild symptoms of headache and hemiparesis to severe manifestations such as a change in the state of consciousness and signs of focal neurological deficits. This variability brings out the umbrella of early neuroimaging for prompt diagnosis. As it is possible to observe from most of the cases that were reviewed, including those by Karageorgiou et al. [6] and Gupta et al. [9], there is a proclivity to present nonspecific symptomatology in brain abscesses such as headache, fever, and hemiparesis. This might bring about some delay in diagnosis that could help explain the relatively high morbidity detected and observed in thalamic abscesses.

Neuroimaging, especially contrast-enhanced CT and MRI scans, essentially remains the cornerstone for diagnosis. All CTs generally show ring enhancement of a lesion in the thalamus, documented in cases presented by, among others, Venger et al. [8] and Mantova and Moya [10]. MRI further delineates the extent of the abscess and, therefore, supports the diagnosis better, as seen in Gupta et al. [9]. However, imaging is usually not decisive in the differentiation between abscesses and other thalamic lesions, such as tumors or infarcts, based on radiological features alone. Very often, stereotactic biopsy or aspiration is required to provide a definitive diagnosis, especially in deep-seated lesions, like those in the thalami.

The most common causative organisms in thalamic abscesses include *Streptococcus* species. More specifically, *S. milleri *and *S. intermedius* are two of the more common species to cause brain abscesses due to their capability to form biofilms. These organisms were frequently isolated in the reviewed cases, including those by Karageorgiou et al. [6] and Mantova and Moya [10]. The prevalence of these pathogens is in keeping with their well-documented tendency to invade deep-seated structures hematogenously, particularly in the context of a remote infection, such as a dental or sinus infection. On the other hand, rare pathogens such as *R. mackenziei* and *H. capsulatum*, as cited by Gupta et al. [9] and Venger et al. [8], respectively, point out the need to look for atypical or fungal organisms, especially in an immunocompromised patient or with a history of environmental exposure. Curiously enough, some patients, like in the case reported by Naidu [23], showed negative cultures upon investigation. These culture-negative abscesses point, once again, to the diagnostic challenges with attempts at isolating the pathogens, with prior antibiotic therapy perhaps suppressing bacterial growth. Molecular techniques, such as polymerase chain reaction (PCR), might increase pathogen detection in such cases; however, this was not one of the common approaches noted in the papers under review.

Considering that surgical intervention was mainly by stereotactic aspiration, this treatment approach was most utilized and highly effective in the majority of cases. Such a minimally invasive technique allows for precise targeting of the abscess while minimizing the risk of damage to critical thalamic structures so vital with the integrating role in motor and sensory information. These include the report by Karageorgiou et al. in 2014 of a 56-year-old male who was treated by stereotactic puncture and drainage followed by intrathecal antibiotics via an Ommaya reservoir [6]. The same resolution of brain abscesses after stereotactic drainage was noted by other authors, including Peker et al. in 2008 and Mantova et al. in 2023 [7,10]. This outlines further the usefulness of the technique in treating deep-seated lesions. Not all cases followed such a straightforward course. For example, Gupta et al., 2024 [9], even went as far as performing additional craniotomy to stereotactic aspiration due to the complexity of the abscess with associated hydrocephalus. It thus depicts the need for an individual approach depending on the extent of the abscess and associated complications. Furthermore, Venger et al. in 1987 [8] and Teixeira et al. in 2010 [15] emphasized that when there is the presence of a fungal abscess, antifungal therapy must be performed; without long-term medication such as amphotericin B, an optimal outcome cannot be achieved.

Apart from surgical drainage, all cases received antibiotic therapy. Most of the patients received broad-spectrum antibiotics like ceftriaxone and metronidazole, which were modified depending on the culture report. In cases involving fungal pathogens, antifungal therapy was administered, and for tuberculous abscesses, antituberculous therapies were enacted, as in the case presented by Mohanty et al. in which antituberculous therapy was initiated after stereotactic aspiration [22]. Complications following surgical intervention were not rare, with hydrocephalus being the most frequent. For instance, Karageorgiou et al. reported intraventricular rupture and cerebellar infarction due to septic emboli as a hazardous complication that required further intervention [6]. Similarly, in the case reported by Gupta et al., the patient required a ventriculoperitoneal shunt for hydrocephalus, and the resulting hemiparesis persisted [9]. This highlights the need for close postoperative follow-up to monitor for complications, especially in patients with large abscesses or significant mass effects.

Despite such complications, the overall long-term outcome in most cases was good. Indeed, Mantova and Moya [10] and Şenol et al. [12] have reported that during the follow-ups, most patients attained full neurological recovery without a residual deficit. Even among the few that presented with complications, such as the development of Holmes' tremor in Peker et al. [7], there was significant improvement with appropriate management, such as deep brain stimulation in this case [7]. Deaths were infrequent, with only two reported among the 36 cases reviewed. Teixeira et al. reported a death due to pulmonary embolism on the 10th postoperative day [15]. Yamamoto et al. also reported one death due to congestive heart failure [19]. Both these deaths were thus due to a medical complication and unrelated directly to the abscess. This indicates that mortality due to a thalamic abscess per se is indeed rare if timely and proper intervention is provided. Follow-up over a period of two months to 10 years showed no recurrences. Thus, thalamic abscesses are apparently curable with the advent of proper surgical and medical management, while the long-term prognosis for the majority of patients remains favorable.

Basyuni et al. reported a thalamic abscess due to a failure to diagnose dental infection as the cause, presenting as an acute-onset dysarthria accompanying left hemiparesis in a 53-year-old patient [31]. Notwithstanding all drainage procedures and subsequent dental clearance, the neurological status of the patient continued to deteriorate, culminating in his eventual demise. The case, therefore, tries to bring out how early diagnosis and timely intervention are essential in the treatment of thalamic abscesses. The brain abscess was a well-recognized source of dental infection, and the prognosis was much better when the source of the primary infection was eradicated early.

Patel et al. emphasized the morbidity concerned with the infections of the area with a case involving the appearance of a thalamic abscess by a *Listeria monocytogenes* abscess in a 71-year-old immunocompromised female [32]. The infection of the central nervous system (CNS) by *L. monocytogenes* is rare, and brain abscesses caused by such kinds of organisms are still rare, comprising only 1-10% of the cases of CNS listerioses. Patel et al.'s patient gradually improved neurologically after a protracted course of intravenous antibiotics [32]. Thalamic abscesses due to *L. monocytogenes *have a very poor prognosis for long-term neurological sequelae and mortality based on the location and rapid improvement of the infection.

Ozgural et al. illustrated that advanced neuronavigation-including magnetic resonance tractography in brain surgery may lower neurosurgical risks in cases involving pediatric thalamic abscesses [33]. In the case reported by them, a five-year-old girl with a congenital cardiac anomaly developed a progressive thalamic abscess. The surgical team engaged neuronavigation along with magnetic resonance tractography during transcranial neuroendoscopic aspiration; this safely evacuated the abscess without damaging vital motor pathways. This case points out the trend of minimally invasive techniques combined with advanced imaging for the management of complex cases of thalamic abscesses. It has been revealed that with the application of the latest surgical armamentarium, such as neuronavigation and endoscopy in the treatment of deep-seated intracranial infections, patients can recover with minimal neurological deficits.

Harizanova et al. present a thalamic abscess that was treated minimally invasively by stereotactic neuronavigation-assisted aspiration [34]. The diagnosis in their case was confirmed, and immediate therapeutic benefit was provided. It serves as an example that sophisticated neuronavigation techniques may be used to precisely localize deep-seated abscesses while preserving the greater safety of the surrounding structures. This is in an effort to outline the importance of combining therapies, surgical and antibiotic, in an attempt to maximize the chances for an optimal outcome since deep-seated abscesses of the thalamus bear higher risks for complications, which may include rupture into the ventricles, with a mortality rate up to 80%. Given the complexity of the infection, timely diagnosis, appropriate surgical intervention, and targeted antibiotic or antifungal therapy when indicated remain keystones in the management of thalamic abscesses. Most importantly, proper and timely interventions reduce complication rates of hydrocephalus and septic emboli. The generally favorable long-term prognosis, with low rates of recurrence, also bears witness to the efficacy of modern neurosurgical and medical modalities for this formidable condition.

## Conclusions

Thalamic abscesses are rare and, due to their deep location and the critical functions of the thalamus in sensory and motor integration, they pose special diagnostic and therapeutic challenges. This systematic review emphasizes variability in clinical presentation, with symptoms often overlapping with other neurological diseases, which can delay diagnosis and contribute to high morbidity. The advances in neuroimaging studies, particularly MRI and CT, have greatly increased the ability for early diagnosis and precise localization of these abscesses, thus helping appropriate intervention. The cases reviewed also hint at the fact that stereotactic aspiration is the most common surgical approach, presenting minimal invasiveness and thus the least amount of insult to surrounding structures. Antibiotic therapy directed at the specific pathogens involved is an important role in the management of the thalamic abscess, particularly in the involvement of *Streptococcus* species. However, hydrocephalus and recurrent infection may be some complications related to it. The long-term prognosis of most patients can be good, with proper management. Mortality rates remained low, and no recurrence was noted in the reviewed cases. Further refinement of diagnostic skills, especially in culture-negative conditions, and fine-tuning minimally invasive surgical techniques for further complication reduction need to be the direction of subsequent studies. The review emphasizes early detection, individualized treatment, and the need for further neuroimaging and neurosurgical advancement in the management of thalamic abscesses.
